# Help-seeking intentions of UK construction workers: a cross-sectional study

**DOI:** 10.1093/occmed/kqae007

**Published:** 2024-02-06

**Authors:** M Duncan, D Bansal, E Cooke

**Affiliations:** Department of Psychology, Institute of Psychiatry, Psychology and Neuroscience, King’s College London, London SE1 1UL, UK; Department of Psychology, Institute of Psychiatry, Psychology and Neuroscience, King’s College London, London SE1 1UL, UK

## Abstract

**Background:**

In response to the high rates of poor mental health in the construction industry, numerous workplace interventions have been designed to address the known and suspected risk factors to employee mental health and well-being. A key challenge of these strategies is low engagement in support services.

**Aims:**

The goals of this research were to investigate the help-seeking intentions of employees in the construction industry, explore levels of mental well-being in this population and provide insight into employee engagement with mental health support strategies.

**Methods:**

Employees from two UK construction companies completed an online cross-sectional questionnaire (*n *= 119), designed to measure help-seeking intentions, levels of mental well-being and worker attitudes towards workplace mental health support strategies.

**Results:**

One-third of the sample reported experiencing an episode of mental health difficulties in the past 6 months. Employees reported a greater preference for seeking support from informal versus formal help sources. Participants were most likely to seek help from a partner and least likely to seek help from a Mental Health First Aider/ Champion. The study also showed some association between help-seeking intention and age of employees.

**Conclusions:**

Given the poor levels of mental well-being in this population, it is essential that adequate workplace support is provided. Whilst formal help sources are important for this population, our study highlights the potential benefits of informal help sources to support employees. Future interventions may therefore wish to consider developing tailored, informal workplace support networks and programmes.

## Teaser

Construction workers are considered an ‘at-risk group’ with higher rates of suicide, stress and mental ill health. We investigated the help-seeking intentions of construction employees to better understand how mental health support strategies are viewed. Findings indicated employees were least likely to seek support from formal help sources, and more general engagement with help strategies was explored. The study points to the benefits of developing informal workplace support networks and programmes to support this occupational group.

**Key learning points**

**What is already known about this subject:**

- Male construction workers are considered at higher risk of suicide, stress and mental ill health than the ‘normal working population’.
- Despite efforts to improve this, rates of suicide and mental ill health have continued to increase among this group.
- Inconclusive evidence surrounding the impact of widely implemented support strategies, such as Mental Health First Aid, suggests that more insight is needed to guide the industry towards better outcomes.

**What this study adds:**

- This study was the first of its kind to investigate UK construction worker’s attitudes towards workplace mental health support strategies and help-seeking intentions.
- Our findings indicate that construction workers prefer informal support and are least likely to engage with a Mental Health First Aider, or other formal help sources, for mental health support.

**What impact this may have on practice or policy:**

- Whilst formal sources of help are important for construction workers, this research points to the benefits of developing additional informal sources of help in the workplace.
- Organizations may wish to consider building informal workplace support capabilities within the construction industry and re-thinking how Mental Health First Aid is implemented and positioned in workplaces.

## Introduction

Mental health (MH) in the construction industry has long been a focus of occupational research and intervention, largely driven by the high rates of suicide observed across the industry globally [1]. In recent years, the industry has seen a surge in occupational interventions that aim to address the known, and suspected, psychosocial risk factors. However, evidence of the impact of such interventions continues to fall behind the rate of implementation. In 2017, the Office for National Statistics reported that the risk of suicide among males working in low-skilled trades, particularly in construction, was three times that of the national average [2]. Between 2015 and 2019, the rate of suicide among this population increased by 50% [3,4]. With construction being one of the industries that were severely disrupted during the coronavirus disease 2019 (COVID-19) pandemic, it is likely that these figures will observe a continued increase.

Given the research findings on this occupational group, it is unsurprising that the construction industry has seen an increase in workplace well-being interventions and policies. These interventions have been designed to improve both individual outcomes (namely MH) and organizational outcomes (such as employee engagement, retention and productivity). Whilst many companies in the construction industry have recorded improvements in organizational outcomes, research to support the impact of organizational well-being interventions on individual outcomes is lacking. Despite this, investment in these strategies continues, and there is a risk that practice has surpassed research, such that organizations are adopting MH and well-being strategies without fully evaluating their efficacy.

One such intervention which has been heavily implemented across multiple sectors, including construction, is Mental Health First Aid (MHFA) [5]. This training is designed to improve MH literacy and the ability to provide in-the-moment support. Whilst the initiative is increasingly popular, the evidence surrounding the effects of MHFA on trainee behaviour and MH outcomes is inconclusive [6,7]. There is also insufficient evidence that MHFA improves the helping behaviours of trainees or the MH of those receiving help [6], or indeed that it has improved the wider management of mental ill health [7]. Consequently, and perhaps in response to the male-dominated nature of the construction industry, with approximately 85% of the workforce being male [8], there has been increasing emphasis on understanding and supporting help-seeking behaviours [9]. This is particularly important given that research consistently shows men have a lower tendency to seek help for MH conditions than women [10].

The primary aim of this paper was to address existing gaps in the evidence base by describing the help-seeking intentions of UK construction workers. The study draws distinctions between different types of help sources, namely formal or informal help. Furthermore, the study sought to explore levels of mental well-being and workplace attitudes in this population. A key objective of this paper was to provide the construction industry with insights into employee engagement with MH support strategies.

## Methods

This cross-sectional study employed an online questionnaire as the primary data collection tool. Ethical approval was obtained from the PNM Research Ethics Panel (Ref: LRU-18/19-9319). Participants were employees from two large construction companies in the UK (>2000 employees) and were recruited through convenience sampling. The Heads of Occupational Health and Safety of the two companies, with whom the researchers had existing collaborations, were briefed on the purpose, methods and timeline for the research, following which they consented to distribute the online questionnaire among their staff. Participation in the study was entirely voluntary and no incentives were provided to complete the questionnaire. Given the nature of this research, no exclusion criteria were applied as it was deemed appropriate to gather the views of all workers in the construction industry. In total, 119 participants commenced the online questionnaire. Data were collected between April and July 2019.

First, participants provided consent and answered demographic questions. Participants then responded to three different psychological measures. The first was the General Health Questionnaire 12-item (GHQ-12) [11], a scale designed to assess levels of psychological well-being. Participants were asked to report how they felt over the past 28 days by responding to each question on a four-point rating scale. The first six items were positively worded (e.g. ‘have you been able to concentrate on whatever you are doing?’) and responses ranged from ‘much less than usual’ (scored as 0) to ‘more so than usual’ (scored as 3). Items 7–12 were negatively worded (e.g. ‘have you lost much sleep over worry?’) and responses ranged from ‘not at all’ (scored as 0) to ‘much more than usual’ (scored as 3). Overall, GHQ scores ranged from 0 (no health condition) to 36 (indication of poor psychological well-being) and were calculated using the GHQ-12 Likert scoring method (0–1–2–3) which is commonly used in research studies, where a lower total score indicates lower psychiatric morbidity. Cronbach’s alpha for this scale demonstrated excellent internal consistency, with α = 0.917.

Participants then completed the Short Warwick–Edinburgh Mental Wellbeing Scale ([S]WEMWBS) [12] which is a condensed 7-item version of the original 14-item WEMBS scale designed to assess mental well-being in non-clinical settings. Items were positively worded statements about thoughts and feelings over the past 2 weeks (e.g. ‘I have been feeling relaxed’), and participants responded to each item on a five-point Likert scale (where 1 = ‘none of the time’ and 5 = ‘all of the time’). Overall mental well-being scores were computed by summing scores across all seven items. Scores ranged from 7 to 35, with higher scores indicating better mental well-being. Cronbach’s alpha for this scale demonstrated good internal consistency, with α = 0.874.

Participants further completed the General Help Seeking Questionnaire (GHSQ) [13], a validated scale that measures intention to seek help for MH issues from a variety of help sources. Participants rated their likelihood of seeking support from 11 different help sources in the event that they experienced a ‘personal or emotional problem’ (P-E) and ‘suicidal thoughts’ (ST). Responses were indicated on a seven-point Likert scale (where 1 = ‘extremely unlikely’ and 7 = ‘extremely likely’), with higher scores meaning a greater likelihood to seek help from the listed source. Overall scores were calculated by grouping the help sources into formal (i.e. MH professional, phone helpline, doctor/GP, MHFA/representative at work), informal (intimate partner, friend outside of work, friend/colleague at work, parent and other relative), would not seek help (‘I would not seek help from anyone’), and other help categories (‘I would seek help from another not listed above’), and generating mean values for each to indicate the sample averages. This analysis was repeated to obtain mean scores for both P-E and ST problem categories.

Finally, participants completed questions of four different organizational measures. These included a three-item measure of job satisfaction (JS); a three-item measure of intention to quit (ITQ); a nine-item measure of organizational commitment (OC) [14]; and a six-item measure of intrinsic job motivation (IJM) [15]. The measures of JS and ITQ were extracted from the Michigan Organizational Assessment Questionnaire [16]. For both the JS and ITQ, participants responded on a seven-point Likert scale (where 1 = ‘strongly disagree’ and 7 = ‘strongly agree’). Total JS and ITQ scores were calculated by averaging the responses for each scale, with higher scores indicating greater JS and ITQ, respectively. Cronbach’s alpha for the JS and ITQ scales demonstrated good and excellent internal consistencies, with α = 0.888 and α = 0.918, respectively.

Participants also completed a nine-item measure of OC. This measure contained three subscales which measured identification, involvement and loyalty with their organization. Responses were recorded on a seven-point Likert scale (where 1 = ‘strongly disagree’ and 7 = ‘strongly agree’), and the total scores were calculated by summing all items together, with higher scores indicating greater OC. Cronbach’s alpha for this scale demonstrated acceptable internal consistency, with α = 0.784. Lastly, participants completed a six-item IJM measure. Responses were recorded on a seven-point Likert scale (where 1 = ‘strongly disagree’ and 7 = ‘strongly agree’). Item responses were summed to give a total score, with higher scores indicating higher IJM.

## Results

One hundred and nineteen participants commenced the survey, with eight respondents providing partial responses. Based on organizational data available, we estimate that this represents a 3% response date. Where missing data occurred, participants were excluded from analyses. Table 1 outlines the demographic details obtained from the sample.

**Table 1.** Demographic characteristics of the sample including gender split, mean age, ethnicities, location of work and weekly working hours

Demographic characteristic	*n* (%)
No. of men	93 (76)
No. of women	26 (24)
Mean (SD) sample age	42 (13.18)
Ethnicities	White (British, Irish and other)—109 (91) Indian—5 (3) Pakistani—1 (1) Bangladeshi—1 (1) Chinese—1 (1) Caribbean—1 (1) Mixed—1 (1)
Location of work	Site-based roles—51 (44) Office-based roles—26 (22) Working in both areas—42 (35)
Mean (SD) weekly working hours	43 (6.76)

Table 2 outlines findings from questionnaire measures including the mean scores and standard deviations of the (S)WEMWBS, GHQ-12, OC, JS, ITQ and IJM for the complete sample, along with gender breakdown of scores.

**Table 2.** Means (*M*) and standard deviations (SD) of questionnaire measures

	Total		Male		Female	
	*M*	SD	*M*	SD	*M*	SD
(S)WEMWBS	24.83^a^	4.40	25.30^b^	4.45	23.04^c^	3.81
GHQ-12	12.96^a^	6.24	12.26^b^	6.10	15.61^c^	6.20
OC	46.54^d^	8.37	46.29^e^	8.53	47.39^c^	7.92
JS	4.76^f^	1.28	4.82^g^	1.26	4.55^c^	1.36
ITQ	3.06^d^	1.79	3.03^h^	1.78	3.15^i^	1.85
IJM	34.84^d^	3.7	35.05^h^	3.80	34.09^i^	3.31

Sample size variations due to difference in response rate: a (*n* = 111), b (*n* = 88), c (*n* = 23), d (*n* = 102), e (*n* = 79), f (*n* = 104), g = (*n* = 81), h (*n* = 80) and i (*n* = 22).

As indicated in Table 2, males had slightly higher mental well-being and lower psychiatric morbidity compared to females in our sample, as demonstrated by the (S)WEMWBS and GHQ-12 scores, respectively. An independent samples *t*-test indicated a significant difference in the GHQ-12 scores for males and females, *t* = 2.34, 95% CI [0.51, 6.19], *P* ≤ 0.05. Similarly, an independent samples *t*-test indicated a significant difference in the WEMWBS scores for males and females, *t* = –2.22, 95% CI [–4.26, –2.44], *P* ≤ 0.05. In line with expectations, we also found a statistically significant negative correlation with a strong effect size between GHQ-12 and (S)WEMWBS scores, with *r*(111) = –0.815, *P* < 0.001, meaning those with higher well-being scores had lower scores of psychiatric morbidity. A significant negative correlation with small effect size was also identified between intention to seek informal help and GHQ scores *r*(111) = –0.242, *P* < 0.05. Further, a significant positive correlation with a small effect size was identified between intention to seek informal help and (S)WEMWBS scores, *r*(111) = 0.247, *P* < 0.05.

With regards to organisational measures, scores on OC and ITQ were marginally higher for females compared to males, with males scoring higher on measures of JS and IJM. However, no significant differences were observed. Lastly, a significant negative correlation was also observed between JS and ITQ, with *r*(100) = –0.673, *P* < 0.001, meaning those with lower job satisfaction were more likely to quit.

Table 3 provides a breakdown of reported MH difficulties as experienced within the past 6 months.

**Table 3.** Reported experience of MH difficulties—*n* (%)

	Yes	No	No response	Total responses
Have you experienced an episode of mental health difficulty over the past 6 months?	41 (32)	65 (50)	24 (18)	130
Have you experienced any suicidal thoughts/feelings over the last 6 months?	13 (10)	92 (71)	25 (19)	130

As outlined in Table 3, around one-third of the sample (*n* = 41) reported that they had experienced an episode of MH difficulties in the past 6 months. Additionally, when asked if participants had experienced any suicidal thoughts/feelings in the past 6 months, 13 (10%) participants said yes, of whom 9 were male and 4 were female. It was interesting to note that around one-fifth of participants actively provided ‘no response’ to this question.

Table 4 reports mean scores on the GHSQ which explores the source of help participants would approach/seek for different problem types. Problems are grouped by problem type, namely P-E and ST and help source (formal, informal, would not seek help and other help source).

**Table 4.** Means (*M*) and standard deviations (SD) of help-seeking (GHSQ) scores

	Overall		P-E		ST	
	*M*	SD	*M*	SD	*M*	SD
Formal	3.02^a^	1.40	2.84^a^	1.48	3.30^b^	1.57
Informal	3.90^a^	1.38	4.08^a^	1.26	3.57^c^	1.66
Would not seek help	3.09^a^	1.83	3.33^j^	2.07	3.05^d^	2.18
Other help	2.06i	1.40	2.02k	1.52	2.16^l^	1.66

Sample size variations due to differences in response rate: a (*n* = 107), b (*n* = 85), c (*n* = 86), and d (*n* = 84), e (*n* = 73), f (*n* = 23), g (*n* = 12), and h (*n* = 13), i (*n* = 103), j (*n* = 104), k (*n* = 101) and l (*n* = 80).

When exploring GHSQ scores, data indicates participants were more likely to seek help from informal rather than formal help sources, regardless of problem type, as shown by the higher mean scores for the total sample in Table 4. A *t*-test indicated that participants would report a preference for informal compared to formal help sources for P-E problems, *t* = 8.11, 95% CI [0.93, 1.54], *P* ≤ 0.001. However, for ST, there was no significant difference in preferences for informal or formal help sources.

When considering gender differences on the GHSQ, males reported less likelihood of seeking help (via formal or informal sources) for any problem type (P-E or ST) compared to females, as indicated by mean scores. However, *t*-tests revealed that these mean differences were not statistically significant. A series of regressions were used to assess the ability of demographic variables to predict help-seeking intentions. In a linear regression model, age was a significant predictor of help-seeking intention. Linear regression identified that age significantly predicted any help-seeking intention. The overall regression was statistically significant, *R*^2^ = 0.58, *F*(1, 101) = 6.24, *P* < 0.014. Interestingly, participants’ likelihood of seeking any help decreased for each 0.19 year of age.

Table 5 reports on scores from the GHSQ in relation to specific help sources within the informal and formal source categories. Responses are listed by problem type (P-E or ST).

**Table 5.** Help-seeking intentions (GHSQ) for P-E and ST problems categorized by sources of help

Help source		Problem type			
		Personal-emotional (P-E)		Suicidal thoughts (ST)	
		*M*	SD	*M*	SD
Informal	Partner	5.67^a^^*^	1.66	5.06^e^^*^	2.07
	Friend	4.46^b^^*^	1.8	3.83^f^^*^	2.04
	Parent	3.49^d^	2.09	3.12^g^	2.20
	Colleague	3.48^c^^*^	1.73	2.73^f^^*^	1.93
	Other relative	3.19^b^^*^	1.97	2.82^f^^*^	2.83
Formal	GP/Doctor	3.31^c^^*^	1.83	3.89^g^^*^	2.09
	Mental health professional^a^	3.15^a^^*^	1.84	3.71^g^^*^	2.08
	Helpline	2.48^b^^*^	1.75	3.17^f^^*^	1.1
	MHFA/Mental health champion	2.33^b^	1.43	2.27^f^	1.55
	Would not seek help	3.33^d^^*^	2.07	3.05^g^^*^	2.18

Sample size variations due to differences in response rate: a (*n* = 107), b (*n* = 106), c (*n* = 105), d (*n* = 104), e (*n* = 86), f (*n* = 83) and g (*n* = 84).

^*^Significant at *P* < 0.05.

^a^‘Mental health professional’ was defined to include ‘Psychologist, counsellor, social worker’.

When exploring all different sources of help, participants reported being most likely to seek help from a partner, followed by a friend for any problem type (i.e. either P-E or ST). Participants indicated being least likely to seek help from a MHFA/ Mental Health Champion for any problem type (P-E or ST), as assessed by mean rank-order values.

## Discussion

This study was an online, cross-sectional questionnaire designed to measure help-seeking intentions, levels of mental well-being and worker attitudes towards workplace MH support strategies among UK-based construction workers. There were four key findings from this study. First, results indicated that one-third of the sample reported experiencing an episode of MH difficulties in the past 6 months. Second, with regards to help-seeking, employees reported a greater preference for seeking support from informal (e.g. peers) versus formal (e.g. GP, counsellor) help sources. Third, findings highlighted a lack of engagement with Mental Health First Aiders, with employees being least likely to seek help from an MHFA/Mental Health Champion for any problem type. Finally, the study showed some association between help-seeking intention and age.

A key strength of this study is that it captured novel data from construction workers, a heavily male-dominated population, on a topic which has been previously difficult to investigate. The topic of MH and help-seeking in this industry has historically been challenging to research, in part due to social norms and ‘stigma’ among construction workers, who are at increased risk of experiencing mental ill health [17]. Our research has not only provided insight into the help-seeking intentions of UK-based construction workers, but also shed light on how current MH support strategies are viewed. Furthermore, our study presented a reasonable sample of employees (*n *= 119) and included construction workers who were working in site and/or office-based locations.

There are, however, limitations to this study. The research was cross-sectional, based on self-report, with data collected from a small number of construction firms in the UK only. Furthermore, there was a higher proportion of males in the sample meaning results should be interpreted with caution. Nevertheless, the gender discrepancy is characteristic of this male-dominated industry [18,19]. Future research may benefit from replication in a larger, more representative sample to validate the findings. Additionally, research may benefit from adopting a longitudinal design to establish whether help-seeking intentions of construction workers are reliable predictors of future engagement in support, and the nature of support that is sought. Indeed, future research may wish to further explore if and how help-seeking intentions precede help-seeking behaviours, and how these may be related to both well-being measures and psychiatric morbidity in the longer term. Finally, research may wish to further explore the aspect of age and perhaps consider targeted interventions for different age groups working in the sector.

When comparing scores from our sample to UK benchmark data, it was interesting to note that scores from the (S)WEMWBS were slightly higher than the general population (mean = 23.7; mean males 23.2; mean females 23.2 [20]), with no significant differences observed. With regards to GHQ-12, examining data from recent UK studies utilizing the Likert scoring method, scores from our sample were higher (12.96 compared to 11.85 [21]), with no significant differences observed. To some extent, our findings therefore further support the wider body of research indicating construction workers are an ‘at-risk group’ with higher rates of stress and mental ill health [2]. Consistent with previous research on help-seeking, employees in our study also indicated a higher likelihood of seeking support from informal rather than formal sources [22,23]. The research also found that women were more likely than men to seek informal support for issues relating to their MH. The findings of this research are broadly consistent with previous research exploring preferences for informal versus formal help-seeking [22], as well as gender differences [24].

The finding that participants reported no significant preference for informal or formal help sources for suicidal thoughts deserves comment. One explanation may be that participants were required to speculate how they would behave in an extreme situation which they had not experienced before. However, reliability analysis of the GHSQ has demonstrated strength in predicting future help-seeking behaviour [13,25], meaning it can be cautiously interpreted that participants’ responses are likely to reflect their future behaviour. Furthermore, the authors acknowledge some ‘missing data’ on question items which were more sensitive in nature, with some participants actively choosing not to complete questions exploring the experience of MH difficulties (see Table 3).

In terms of individual sources of help, participants in this study reported being most likely to seek support from a partner and least likely to seek help from an MHFA/Mental Health Champion. As such, findings confirm that employees express a preference for seeking help from those within their social networks for MH issues [26]. These results question the utility of current interventions in place in the industry, specifically MHFA, and offer some justification for future strategies aimed at promoting informal support strategies as a workplace MH and well-being intervention, such as those demonstrated by the ‘Mates in Construction (MATES) Australia’ programme [27,28]. Added to this, contemporary research has demonstrated the benefits of using smartphone applications to improve suicide prevention literacy and help-seeking intentions [29], which is an area of research that warrants further investigation.

To conclude, this research has provided novel insight into help-seeking intentions among UK construction workers. Whilst formal sources of help are important for construction workers, this research points to the benefits of developing additional informal sources of help in the workplace, and questions the utility of MHFA as a source of support. Organizations may therefore wish to consider building informal support capabilities within construction and re-think how MHFA is implemented and positioned in workplaces.
